# *Ureaplasma urealyticum* infection presenting as altered mental status in a post-chemotherapy patient: Case report and literature review

**DOI:** 10.3389/fmed.2022.1057591

**Published:** 2022-11-25

**Authors:** Eunice J. Y. Kok, Y. L. Lee

**Affiliations:** Division of Anesthesiology, Singapore General Hospital, Singapore, Singapore

**Keywords:** *Ureaplasma urealyticum*, hyperammonemia, altered mental status (AMS), immunocompromised, chemotherapy, cancer

## Abstract

Hyperammonemia due to Ureaplasma infection is rare but often fatal, largely due to the delayed recognition, diagnosis, and treatment of the condition. It has mostly been described in solid organ transplant patients in the literature. This case presents the diagnostic challenge of an immunocompromised patient with previous resected pancreatic head adenocarcinoma and chemotherapy, presenting with altered mental status due to hyperammonemia from Ureaplasma infection. It is imperative to consider this condition in unexplained hyperammonemia, especially in immunocompromised patients. Timely diagnosis of this condition can help to reduce complications from encephalopathy such as cerebral edema and seizures.

## Introduction

Ureaplasma infection is a rare cause of non-cirrhotic hyperammonemia. The literature surrounding this topic is largely limited to case reports and series. It has been traditionally described in solid organ transplant recipients, but there has been increasing numbers of reports on this in immunocompromised, non-transplant patients (1–3). Here, we describe the diagnostic challenge of a case of hyperammonemia syndrome due to Ureaplasma infection in a post-chemotherapy patient, discuss our approach to altered mental status (AMS), and review the available literature on hyperammonemia syndrome due to Ureaplasma infections. Written consent has been obtained from the patient’s relative in accordance with Singapore’s research ethics guidelines.

## Case description

The case is a 67-year-old female with hypertension, hyperlipidemia, and left breast cancer for which she underwent a mastectomy and adjuvant chemotherapy from 2016 to 2017. In July 2020, she was diagnosed with a second malignancy—locally advanced pancreatic head adenocarcinoma, received neoadjuvant chemotherapy from August 2020 to January 2021, surgical resection (total pancreatectomy, extended right hemicolectomy and portal vein resection) in May 2021, and adjuvant chemotherapy from 18 October 2021 to 31 October 2021.

In December 2021, she attended a routine clinic review and was found to be significantly malnourished—she had extremely poor oral intake, wasting of muscle bulk, a body mass index of 18.9, and severe hypoalbuminemia (albumin 15 g/L; reference range 40–50 g/L). Her vital signs were stable—temperature of 36.2 degrees Celsius, blood pressure was 103/67 mmHg, heart rate of 91 beats per minute, and SpO2 96% on room air. She was admitted for inpatient enteral nutrition support. She remained well for a week before developing acute AMS. Clinical examination revealed a gradual decline of her Glasgow Coma Scale (GCS) from 15 to 11 over several days, but was otherwise unremarkable with no focal neurological deficits.

An extensive biochemistry panel revealed hyperammonemia (with an initial ammonia level of 144 umol/L; reference range 16–53 umol/L), hypophosphatemia (0.57 mmol/L; reference range 0.94–1.50 mmol/L), and micronutrient (zinc, copper, selenium, vitamin A, vitamin D2, vitamin D3, vitamin E) deficiencies. There was suspicion of an acute coronary syndrome in view of new anterolateral ST segment elevations on her electrocardiogram and raised troponin levels, but this was ruled out with a negative coronary angiogram and attributed to stress cardiomyopathy with a depressed ejection fraction of 25%. Neuroimaging (computed tomography and magnetic resonance imaging of brain, magnetic resonance angiogram of brain) was normal. An electroencephalogram showed severe diffuse encephalopathy with generalized triphasic waves suggestive of metabolic encephalopathy. Basic microbiological investigations including aerobic and anaerobic blood cultures, she was initially managed for metabolic encephalopathy, likely contributed by constipation, hyperammonemia, electrolyte imbalance, and stress cardiomyopathy. Her mental status improved to normal with regular opening of bowels and electrolytes correction.

However, her neurological status gradually deteriorated from a GCS of 15 to 3 over the next 2 weeks, associated with multiple episodes of hypoglycemia. There was no improvement to her mental status after correction of hypoglycemia. Thus, a repeat workup including neuroimaging, electroencephalogram and lumbar puncture was performed. A comprehensive panel of microbiological investigations---including aerobic and anaerobic bacterial blood cultures, fungal blood cultures, viral serology panel,^1^ sputum cultures, urine cultures, cerebrospinal fluid (CSF) cultures, CSF meningoencephalitis panel,^2^ tests for syphilis infection and tuberculosis infection—was also sent. The investigations were significant for hyperammonemia (234 umol/L), positive urine cultures for *Escherichia coli*, and positive sputum cultures for *Klebsiella pneumoniae* and *Stenotrophomonas maltophilia*. She was started on intravenous (IV) Meropenem and Minocycline to cover for these organisms, and concomitantly worked up for the underlying etiology of hyperammonemia.

A liver duplex ultrasound revealed possible right portal vein thrombosis. Hence, she underwent a percutaneous transhepatic biliary drainage, portal vein angioplasty and stenting. Her ammonia levels improved transiently to 74 umol/L but rose back up again after several days post-procedure with no improvement in neurology. Her blood and endotracheal aspirates were sent for Ureaplasma and Mycoplasma cultures. She had earlier been commenced on a course of Minocycline (IV Minocycline 200 mg loading dose, followed by 100 mg every 12 h for a week) to cover for the Stenotrophomonas pulmonary infection, as well as for empirical coverage of possible Ureaplasma infection. The blood culture eventually returned positive for *Ureaplasma urealyticum*. By this time, she had completed a week of Minocycline, and was continued on another week of Azithromycin (Oral Azithromycin 500 mg once daily). A repeat Ureaplasma blood culture thereafter was negative. The patient had undergone chemotherapy with *TS-One* (Tegafur, Gemeracil, Oteracil) in October 2021. However, her medical oncologist felt that the time frame (*TS-One* was given more than 6 weeks before her presentation of hyperammonemia) was not compatible with *TS-One* as the cause of her current encephalopathy or hyperammonemia. Finally, a metabolic panel for urea cycle disorders was sent and returned negative.

Simultaneously, supportive treatment was initiated to promote ammonia clearance with lactulose, rifaximin, sodium benzoate, and hemodialysis, as well as to reduce ammonia production through dietary protein restriction.

Retrospectively, we found that her mentation improved most dramatically with a combination of: (1) lowering her ammonia levels with dialysis, (2) treatment of Ureaplasma infection with the appropriate anti-microbials, (3) treatment with high dose thiamine for presumed thiamine deficiency, and (4) nutritional replacement for micronutrient deficiencies. The final diagnosis was metabolic encephalopathy contributed by thiamine deficiency (causing a Wernicke-like state), exacerbated by hyperammonemia from Ureaplasma infection, and micronutrient deficiencies related to a post-pancreatectomy state. Subsequently, with the appropriate treatment and normalization of ammonia levels, she recovered to full neurology and was able to follow instructions. However, her protracted intensive care unit (ICU) stay was complicated by critical illness myopathy. She was discharged from ICU after 41 days but eventually succumbed to nosocomial infections and passed on day 83 of her hospital stay.

Table 1 shows the timeline of events that occurred.

**TABLE 1:** Timeline of events.

Date	Events
December 23, 2021	● Found to be significantly malnourished at a routine clinic review ● Admitted for inpatient enteral nutrition support
January 1, 2022	● Developed altered mental status ● Biochemistry panel sent for evaluation of altered mental status ⇒ Hyperammonemia (144 umol/L), hypophosphatemia, micronutrient deficiencies ⇒ Coronary angiogram done in view of new anterolateral ST segment elevations on ECG and raised troponin levels ● Coronary angiogram negative for significant coronary vessel occlusion ⇒ Diagnosed with stress cardiomyopathy with a depressed ejection fraction of 25%
January 13, 2022	● GCS gradually dropped from 15 to E3V5M3 ● Hypoglycemia (capillary blood glucose 2.0 mmol/L) detected and corrected, but no improvement in mental status
January 14, 2022	● CT brain performed for altered mental status and worsening GCS—normal ● Developed hospital-acquired pneumonia and urinary tract infection, started on broad-spectrum IV antibiotics
January 18, 2022	● GCS dropped further to 3 ● EEG performed for altered mental status and worsening GCS—severe diffuse encephalopathy with generalized triphasic waves suggestive of metabolic encephalopathy
January 19, 2022	● MRI brain performed for altered mental status and worsening GCS—normal
January 21, 2022	● Developed type 2 respiratory failure from severe hospital-acquired pneumonia ⇒ Intubated and sent to ICU ● Initial impression was septic encephalopathy secondary to bilateral hospital-acquired pneumonia ● Worsening hyperammonemia—peak 234 umol/L ● Started on hemodialysis for ammonia clearance
January 22–30, 2022	● No improvement in mental status despite adequate treatment of hospital-acquired pneumonia ● Ureaplasma blood culture sent on January 27, 2022, started on empirical Minocycline
January 31, 2022	● Repeat biochemistry panel, EEG and neuroimaging done—significant for hyperammonemia and severe diffuse encephalopathy likely from metabolic causes ● Metabolic panel for urea cycle disorders sent—normal ● Liver duplex ultrasound performed for evaluation of possible shunt as a cause of hyperammonemia—possible right portal vein thrombosis
February 1, 2022	● Underwent a percutaneous transhepatic biliary drainage (PTBD) and portal vein angioplasty and stenting for possible portal vein thrombosis ● Ammonia levels improved transiently to 74 umol/L after the procedure, but rose back up again ● No improvement in mental status
February 4, 2022	● Lumbar puncture performed—CSF biochemistry panel normal, CSF cultures normal, syphilis screen negative, autoimmune encephalitis panel normal, paraneoplastic panel normal ● Mental status started to improve
February 8, 2022	● First Ureaplasma blood culture sent on January 27, 2022 returned as positive ● 1 week of Minocycline completed ● Second Ureaplasma blood culture sent ● Started on Azithromycin
February 9–March 14, 2022	● Mental status continued to improve, gradually returned to her normal baseline ● Suffered a series of nosocomial and opportunistic infections—intra-abdominal sepsis from PTBD leak and cytomegalovirus colitis, catheter-associated urinary tract infection, candidemia
March 15, 2022	● Developed acute respiratory failure with hemodynamic instability from new severe pneumonia and fluid overload ● Passed on day 83 of hospital stay

## Discussion

This case demonstrated the diagnostic challenge of a patient with a background resected pancreatic head adenocarcinoma and previous chemotherapy, presenting with AMS. After an extensive evaluation, she was found to have hyperammonemia syndrome secondary to Ureaplasma infection. To our knowledge, this is first reported case of hyperammonemia syndrome due to Ureaplasma infection presenting in a post-chemotherapy patient (the only other similar case being a patient undergoing chemotherapy). This case adds to the growing amount of literature and interest in this topic, especially in non-transplant patients, and emphasizes the post-chemotherapy, immunocompromised state as a plausible risk factor for disseminated Ureaplasma infections. Certainly, this will require further reports of similar cases and a more rigorous study methodology to test the conceivability and validity of this hypothesis.

### Evaluation of altered mental status

**FIGURE 1:**
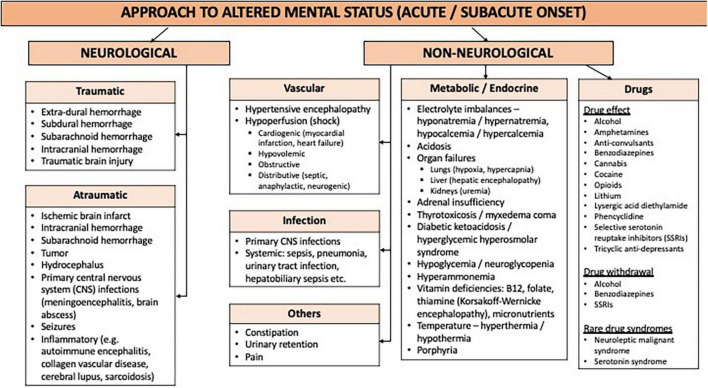
Approach to altered mental status (AMS) (acute and subacute onset).

Altered mental status is a broad presentation with a myriad of differential diagnoses. The first step (shown in Table 2) is to differentiate between an acute or subacute disease, and chronic cognitive decline. Here, we endeavor to create our systematic approach to this clinical presentation with a focus on the acute and subacute etiologies (refer to Figure 1).

**TABLE 2:** Step 1 in the approach to altered mental status (AMS)—acute or subacute disease vs. chronic cognitive decline.

	Delirium	Dementia	Depression
Onset	Acute, hours to days	Insidious, chronic, months to years	Variable
Course/Progression	Fluctuating	Progressive	Variable
	Sun-downing	Sun-downing	
Orientation	Disoriented	Oriented until later stages	Usually oriented
Attention	Inattentive	Attentive	Inattentive
Speech and language	Incoherent, illogical	Aphasia, anomia	Normal
Affect/Mood	Dysphoric, labile	Depressed, abulic	Depressed
Sleep	Disturbed sleep-wake cycle	Reversed sleep-wake cycle	Early morning awakening

History taking is often limited from patients with AMS, hence it is prudent to obtain collaborative history from the patient’s relatives and caregivers. A detailed drug chart including over-the-counter medications, traditional medications and other possible use of illicit substances is also essential. Physical examination begins with an assessment of the patient’s airway, breathing, and circulation, followed by a full neurological examination to check for focal neurological deficits. Additionally, an assessment of the perfusion status, looking for signs of a prior trauma, localizing signs of infection, and signs of organ failures can provide relevant and valuable information.

Point-of-care testing is simple, convenient, and very useful in the evaluation. This includes a fingerstick test for capillary blood glucose to check for hypoglycemia, and an arterial blood gas to check for respiratory failure and metabolic derangements.

The choice of laboratory and radiological investigations should be guided by the clinical picture obtained from history taking and physical examination. Table 3 shows a non-exhaustive list of investigations.

**TABLE 3:** Investigations for the workup of altered mental status (AMS).

Laboratory	Radiological
● Full blood count ● Biochemistry panel—electrolytes (sodium, potassium, calcium, phosphate), glucose, urea, creatinine, bicarbonate ● Liver function test ● Arterial blood gas ● Thyroid function test ● Ammonia ● Thiamine ● Serum B12 and folate ● Micronutrients (zinc, copper, selenium, vitamin A, vitamin D2, vitamin D3, vitamin E) ● Syphilis panel ● Blood and urine toxicology ● Urinalysis ● Lumbar puncture (opening pressure, CSF glucose and protein, CSF white blood cells and red blood cells, gram stain and culture, acid fast bacilli smear and culture, fungal microscopy, meningitis multiplex/tetraplex panels, cytology, oligoclonal band) ● Autoimmune encephalitis panel ● Serum paraneoplastic panel	Neuroimaging ● CT brain ● MRI brain and angiogram Systemic infection ● Chest X-ray ● CT thorax, abdomen, pelvis **Others** ● Electroencephalogram ● Electrocardiogram

### Hyperammonemia in Ureaplasma infections

Ureaplasma species is commonly found as a urogenital commensal and typically has low pathogenicity (4). However, two specific organisms *Ureaplasma urealyticum* and *Ureaplasma parvum* have been increasingly reported to cause disease, most commonly in the form of hyperammonemia syndrome. Hyperammonemia syndrome is characterized by high serum ammonia concentrations [there is no defined level but some suggest an upper limit of 200 umol/L (5)] with new onset or progressive neurological dysfunction (1, 5).

Ureaplasma contains urease, an enzyme which hydrolyzes urea to ammonia and carbon dioxide. This hydrolysis reaction provides a potential gradient to generate energy for the organism in the form of adenosine triphosphate (6). Simultaneously, the ammonia product from the hydrolysis reaction becomes a substrate for the synthesis of more urea, thereby perpetuating this cycle and the growth of the Ureaplasma organism (7). It is widely postulated that the eventual build-up of ammonia to supernormal levels causes neurological dysfunction from metabolic encephalopathy due to hyperammonemia, which can lead to fatal complications such as cerebral edema and brain herniation (1).

While hyperammonemia syndrome was described almost three decades ago by Davies et al. (8) it was not until Bharat et al.’s landmark paper (7) in 2015 that there was a postulated link between Ureaplasma infection and hyperammonemia. In a case series in lung transplant patients, Bharat described several patients who had unexplained, fatal hyperammonemia syndrome. This was eventually attributed to Ureaplasma infection, after Ureaplasma organisms were isolated in the bronchoalveolar lavage cultures and blood cultures of both the donors of the affected recipients, and the affected recipients. Since then, there have been several other case reports on hyperammonemia from Ureaplasma infections. A systematic review and meta-analysis on hyperammonemia syndrome associated with Ureaplasma spp. infections in immunocompromised patients and transplant recipients suggested that there was a higher incidence of hyperammonemia syndrome in Ureaplasma-positive lung transplant recipients (41.67%) as compared to Ureaplasma-negative recipients (2.84%) (1). The affected population is mostly solid organ transplant recipients (lung and kidney), with a few reported cases on immunocompromised patients such as those with hematological malignancies (9, 10), those undergoing hematopoietic stem cell transplant (3) and those undergoing chemotherapy (11).

The clinical presentation of hyperammonemia from Ureaplasma infection is usually neurological dysfunction—it may present as AMS, agitation, disorientation, lethargy, confusion, drowsiness, and a drop in the GCS, or seizures (3). Laboratory investigations are often unremarkable apart from hyperammonemia with normal neuroimaging. Electroencephalograms may show diffuse encephalopathy typical of metabolic encephalopathy.

Hyperammonemia from Ureaplasma infections has been described in several reports to be fatal without prompt recognition, diagnosis and treatment with a mortality rate in immunocompromised and post-transplant patients of 42–75% (12). Hence, there should be a high index of suspicion for Ureaplasma infection this subgroup of patients and investigation performed. Hyperammonemia from Ureaplasma infections is notorious for not responding well to usual ammonia-lowering strategies without the appropriate anti-microbial treatment for the Ureaplasma infection (13). As Ureaplasma species lack a cell wall, they are unable to be visualized with gram stain nor cultured in conventional medium (13), and hence require a special culture medium for growth and identification. This may take several days to a week to be out and so empirical use of antibiotics to cover for Ureaplasma have been recommended so as not to delay treatment which could result in major morbidity and mortality (11, 13). Some centers offer molecular testing for Ureaplasma species in the form of polymerase chain reaction (PCR) assays. There are two types of PCR assays—gel-based conventional PCR assays which use targeted sequences of 16s ribosomal ribonucleic acid found in Ureaplasma species, and real-time PCR assays which identify urease genes and their subunits (14). These PCR assays have a turnaround time of a few hours—significantly faster than that for the Ureaplasma culture.

There are two main tenets in the management of this syndrome. First, treat the underlying cause (Ureaplasma infection) with the appropriate anti-microbials. As Ureaplasma species lack a cell wall, they do not respond well to beta-lactams. As such, antibiotics such as macrolides, tetracyclines, and fluoroquinolones are recommended (6, 15). There is some debate about whether a single agent or combination therapy (with more than one agent from different classes) is a better option for treatment—no high-quality, conclusive evidence has been presented thus far. In our patient, a single agent (Minocycline) seemed to have been sufficient for eradication of the Ureaplasma bacteremia. The second agent (Azithromycin) was started after the second Ureaplasma blood culture was taken, with the intent of not delaying treatment should the culture return as positive for Ureaplasma a week later. The second Ureaplasma blood culture turned out to be negative. If there are infected collections, surgical drainage should be considered for source control, in addition to anti-microbial treatment (15).

Second, lowering the serum ammonia with strategies to promote ammonia clearance and to reduce ammonia production. Lactulose acidifies the gut, and causes ammonia to be ionized into ammonium, which reduces the absorption and increases the excretion of ammonia from the gastrointestinal tract (16). Rifampicin is an anti-microbial used to reduce ammonia-producing bacteria (17, 18). Dietary modifications can be made to reduce the protein load so as to reduce the substrate for ammonia production (16). In our patient, the protein content in her diet was restricted to 0.8 g/kg/day, and was gradually increased back to 1.2–1.5 g/kg/day with improving ammonia levels. Nitrogen scavengers such as sodium benzoate and sodium phenylbutyrate promote the diversion of nitrogen away from the urea cycle, to other metabolic pathways (18). Finally, hemodialysis may be indicated for hyperammonemia that is refractory to medical therapy. Ammonia is a small molecule, water soluble and not highly protein bound—these intrinsic qualities allow it to be efficiently cleared by hemodialysis. Higher efficiency dialysis (with higher blood flow rate, higher dialysate flow rate, and larger dialyzer membrane surface area) results in better ammonia clearance (19), and has been associated with increased survival in post-lung transplantation patients with hyperammonemia syndrome (20).

## Conclusion

Ureaplasma species infections should be considered in patients with unexplained hyperammonemia, and particularly in immunocompromised patients (such as patients who are undergoing or have undergone systemic chemotherapy) who are more susceptible to disseminated infections. Given the high mortality of untreated hyperammonemia syndrome from Ureaplasma infections, prompt recognition, diagnosis and treatment are the cornerstones in the management of this condition. It is important to consider empirical antibiotics for Ureaplasma infections before the Ureaplasma culture or PCR results are out. The increasing awareness about this condition in the medical and scientific community is encouraging and a big step forward in reducing major morbidity and mortality from this treatable condition.

## Data availability statement

The original contributions presented in this study are included in the article/supplementary material, further inquiries can be directed to the corresponding author.

## Author contributions

EK helped to conceptualize and write the manuscript. YL helped to conceptualize and review the manuscript. Both authors contributed to the article and approved the submitted version.

## Footnotes

1 The viral serology panel included evaluation for the following organisms: Hepatitis B, Hepatitis C, HIV, JC virus, Cytomegalovirus, Herpes Simplex virus, Varicella-Zoster virus.

2 The CSF meningoencephalitis panel included evaluation for the following organisms: *Escherichia coli*, *Hemophilus influenzae*, *Listeria monocytogenes*, *Neisseria meningitidis*, *Streptococcus agalactiae*, *Streptococcus pneumoniae*.
